# Interaction of Bioactive Coomassie Brilliant Blue G with Protein: Insights from Spectroscopic Methods

**DOI:** 10.3797/scipharm.1008-15

**Published:** 2010-11-06

**Authors:** Umesha Katrahalli, Shankara Sharanappa Kalanur, Jaladappagari Seetharamappa

**Affiliations:** Department of Chemistry, Karnatak University, Dharwad 580 003, India

**Keywords:** Coomassie brilliant blue G, Physiological condition, Optical spectroscopy, Binding parameter, Thermodynamic parameter

## Abstract

The binding of coomassie brilliant blue G (CBB) to bovine serum albumin (BSA) was investigated under simulative physiological conditions employing different optical spectroscopic techniques *viz.*, fluorescence emission, UV–visible absorption and FTIR. Fluorescence quenching data obtained at different temperatures suggested the presence of dynamic type of quenching mechanism. The binding constant of CBB-BSA and the number of binding sites (n) for CBB in BSA were calculated and found to be 4.20 × 10^4^ M^−1^ and 0.96 respectively, at 302 K. The value of n close to unity indicated that the protein has a single class of binding sites for CBB. The thermodynamic parameters revealed that the hydrophobic forces played a major role in the interaction of CBB to BSA. The distance between the CBB and protein was calculated using the theory of Föster’s Resonance Energy Transfer (FRET). The conformational change in the secondary structure of BSA upon interaction with dye was investigated by synchronous fluorescence and FTIR techniques. Competitive binding studies were also carried out to know the location of binding of CBB on BSA.

## Introduction

**Fig. 1.**
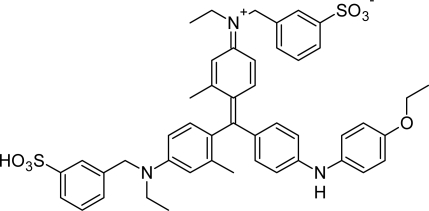
Structure of Coomassie brilliant blue G.

Coomassie brilliant blue G (CBB) dye (Fig. 1), [also known as coomassie dye] is employed to stain proteins in sodium dodecyl sulfate and blue native polyacrylamide gel in electrophorosis gels. It is used in the quantification of electrophoretically separated protein [1]. The CBB has also been popularly used in biochemical and clinical laboratories for the purification and quantification of proteins [2]. Recently, CBB was used in the treatment of spinal injuries in rats [3]. It was also used as a stain to assist surgeons in retinal surgery [4].

Serum albumin (SA), often simply referred as albumin, is the most abundant plasma protein in humans as well as in other mammals which plays a major role in the transport and deposition of many drugs in the blood. This albumin is essential for the maintenance of osmotic pressure which is needed for proper distribution of body fluids between intravascular components and body tissues. SA consists of three homologous domains (I–III), each of them is composed of two subdomains (A and B) [5, 6]. In the IIA and IIIA pocket, the majority of small ligands like drugs are bound and carried to the target site [7–10]. Therefore, it is important to study the interaction of the drug with the serum albumin, which plays an important role in pharmacology, pharmacokinetics and pharmacodynamics. In this regard, bovine serum albumin (BSA) has been studied extensively, partly because of its structural homology with human serum albumin (HSA) [11].

In the present report, we have focused on the interaction of CBB with bovine serum albumin and obtained the information with regard to binding constant, number of binding sites, thermodynamic parameters, and conformational changes in the protein employing spectroscopic techniques. Among these, fluorescence technique is a well known practical method for studying protein interactions with various ligands [12–14] as it yields a vast amount of information on binding characteristics and the microenvironment surrounding the protein residues.

## Materials and Methods

### Materials

Bovine serum albumin (fatty acid free, fraction V) and Brilliant blue G–250 were purchased from Sigma Chemicals Co. Millipore water was used throughout the experiment. All other chemicals used in the present study are of analytical reagent grade.

### Equipment and spectral measurements

The fluorescence spectra were recorded on a spectrofluorimeter model F–7000 (Hitachi, Japan) equipped with a 1.0 cm quartz cell and a thermostat bath. Inner filter effect was not observed during fluorescence measurements. Necessary corrections have been made. The widths of both excitation and emission slit were set to 5 nm in the experiment. The UV–vis spectra were recorded on a double beam CARY 50–BIO UV–vis spectrophotometer (Varian, Australia) equipped with a 1.0 cm quartz cell and a slit width of 5 nm. FTIR spectra were acquired on a Thermo Nicolet–5700 FTIR spectrometer (Waltham, MA, USA).

### Procedures

The fluorimetric titrations were carried out and the fluorescence intensities of protein were recorded at around 340 nm upon excitation at 296 nm. Based on preliminary studies, the concentration of BSA was fixed at 2.5 μM while that of the dye was varied from 2.5 to 25 μM. The interaction studies were carried out at 293, 302 and 309 K.

The site probe studies were performed using different probes viz., warfarin, ibuprofen and digitoxin respectively for site I, II and III by keeping the concentration of both protein and the probe constant (each at 2.5 μM) and varying the concentration of the dye from 2.5 to 25 μM.

The absorption spectra were recorded by scanning the binary mixture of dye and the protein in the wavelength range of 250 to 300 nm.

The FTIR spectrum of BSA in the absence of CBB was obtained by subtracting the IR spectrum of buffer. The FTIR spectrum of BSA in presence of CBB was obtained by subtracting the IR spectrum of free CBB in buffer from that of the bound CBB to protein in the range of 1500 to 1700 cm^−1^.

## Results and discussion

### CBB–induced quenching studies of bovine serum albumin

**Fig. 2.**
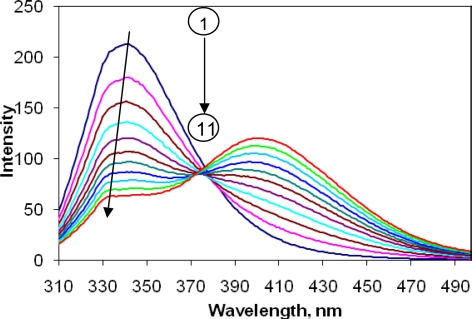
Fluorescence spectra of BSA. Concentration of BSA was fixed at 2.5 μM (1) and that of CBB was varied in the range of 2.5 to 25 μM (2–11).

The fluorescence of SA mainly resides in the emission from the tryptophan, Trp (∼ 340 nm) and tyrosine, Tyr (∼ 315 nm) residues. Fluorescence spectra of BSA were recorded in the presence and absence of CBB upon excitation at 296 nm. The fluorescence emission of protein was observed to be quenched (around 340 nm) in a concentration dependent manner by CBB (Fig. 2).

Quenching of the intrinsic fluorescence of protein can be used to retrieve information on ligand–protein binding. The fluorescence quenching data were analyzed using the Stern–Volmer equation shown below [15]:

Eq. 1. $$\frac{F_{0}}{F}=1+K_{SV}[Q]$$

where *F*_0_ and *F* are the steady–state fluorescence intensities in the absence and presence of the quencher (CBB), respectively; Q is the quencher concentration and *K_SV_* is the Stern–Volmer quenching constant.

**Fig. 3.**
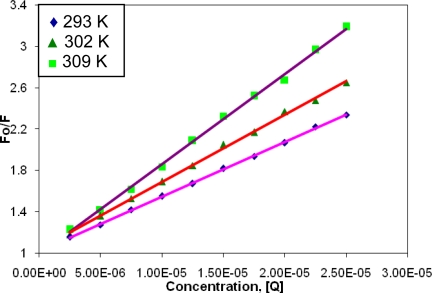
The Stern–Volmer plot for quenching of BSA.

The plot of the fluorescence intensity ratio of BSA in the absence and presence of quencher (CBB) as a function of the quencher concentration showed a linear dependence (Fig. 3). The values of *K_SV_* for BSA–CBB system were calculated from the slope of the Stern–Volmer plot and the corresponding values are given in Table 1.

The quenching mechanisms are usually classified as either dynamic quenching or static quenching. Dynamic and static quenching can be differentiated by their differing dependence on temperature [15]. From the results of Stern–Volmer plots, we noticed that the *K_SV_* values increased with rise in temperature indicating the presence of dynamic quenching mechanism in the interaction of the protein with CBB. Further, it was also noticed that the fluorescence intensity of BSA was decreased in the presence of CBB with a slight blue shift of the maximum emission wavelength indicating that the chromophores of the protein were placed in a more hydrophobic environment after the addition of CBB. The blue shift could also be explained by a preferential quenching of the Trp residues that would leave only the Tyr residues to contribute to protein fluorescence. Moreover, we observed a concomitant increase in the fluorescence intensity at 401 nm, which is the characteristic wavelength of the bound BSA. This phenomenon might be the result of the radiationless energy transfer between CBB and BSA. Further, the existence of an isoactinic point at 375 nm indicated the presence of bound and unbound CBB at equilibrium.

### Determination of binding constant and binding capacity

CBB induced fluorescence quenching data of BSA was analyzed to obtain the binding parameters like binding constant (*K*) and the number of binding sites (*n*) from the equation shown below [16, 17]:

Eq. 2. $$\text{log}\left(\frac{F_{0}-F}{F}\right)=\text{log}K+n\text{log}[Q]$$

**Fig. 4.**
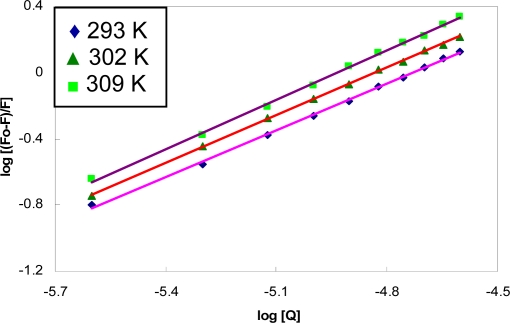
The plot of log[(F_0_–F)/F] versus log[Q] for quenching of BSA by CBB at different temperatures.

Fig. 4 depicts the linear plot of *log* [(*F*_0_–*F*)/*F*] *versus log* [Q] and the corresponding results of *K* and *n* are given in Table 1. The slope registered in this plot was noticed to be close to unity revealing that one ligand molecule bound to a molecule of protein. This means, BSA has a single class of binding site for CBB. Hence, we propose that CBB most likely binds to the hydrophobic pocket located in subdomain IIA, that is to say, Trp–214 is near or within the binding site.

### The interacting force between CBB and protein

There are essentially four different types of interacting forces, *viz*., hydrogen bonds, van der Waals forces, electrostatic forces and hydrophobic interactions, which could play a major role in ligand binding to serum albumins. The signs and magnitudes of thermodynamic parameters determine the nature of interacting forces, which are taking part in drug–protein interactions [18]. The Δ*H*^0^ and Δ*S*^0^ were calculated using the van’t Hoff’s equation shown below [19]:

Eq. 3. $$\text{log}K=\frac{-\Delta H^{0}}{2.303\;\text{RT}}+\frac{\Delta S^{0}}{2.303\;R}$$

The values of Δ*G*^0^ for the binding process at different temperatures were calculated using the equation shown below and the corresponding results are given in Table 1.

Eq. 4. $$\Delta G^{0}=-2.303\;\text{RT}\;\text{log}\;K$$

The negative values of Δ*G*^0^ indicated spontaneity of the binding process. The positive values of both Δ*H*^0^ and Δ*S*^0^ observed in CBB–BSA system showed revealed that the hydrophobic forces played a major role in the binding process between CBB and BSA [18].

### Exchange of energy between protein and dye

The fluorescence quenching of BSA by CBB revealed the occurrence of energy transfer between the protein and dye. According to the Förster’s non–radiative energy transfer theory [15, 20], the energy transfer between protein and dye depends on (*a*) the overlap of the fluorescence emission spectrum of the donor with UV–vis absorption spectrum of the acceptor and (b) the distance of approach between the donor and acceptor. The efficiency, E of a FRET process depends on the inverse sixth–distance between donor and acceptor (*r*) as well as on the critical energy transfer distance or Förster radius (*R*_0_) under the condition of 1:1 situation of donor to acceptor concentrations and can be expressed by the following equation:

Eq. 5. $$E=1-\frac{F}{F_{0}}=\frac{R_{0}^{6}}{R_{0}^{6}+r^{6}}$$

where *E* is the efficiency of energy transfer; *F* and *F*_0_ are the fluorescence intensities of the donor in the presence and absence of the acceptor, respectively. The critical distance, *R*_0_ when the transfer efficiency is 50% can be calculated using the equation:

Eq. 6. $$R_{0}^{6}=8.8\times 10^{-25}k^{2}\Phi N^{-4}J$$

where *k*^2^ is the spatial orientation factor of the dipole, *Φ* is the fluorescence quantum yield of the donor, *N* is the refractive index of the medium and *J* is the overlap integral of the fluorescence emission spectrum of the donor and the absorption spectrum of the acceptor.

The term *J* is expressed as;

Eq. 7. $$J=\frac{\sum F(\lambda )ɛ(\lambda )\lambda^{4}\Delta \lambda }{\sum F(\lambda )ɛ(\lambda )}$$

where *F*(*λ*) is the fluorescence intensity of the donor at wavelength *λ* and *ɛ*(*λ*) is the molar absorptivity of the acceptor at wavelength *λ*.

**Fig. 5.**
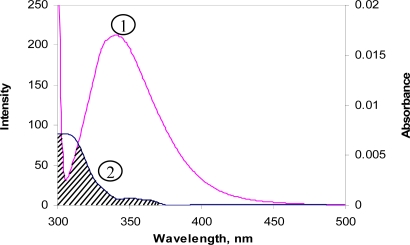
The overlap of BSA fluorescence spectrum (1) and CBB absorption spectrum (2). [BSA] : [CBB] = 1 : 1.

Fig. 5 depicts the overlap between the fluorescence emission spectrum of free protein and the absorption spectrum of dye. The efficiency of energy transfer and overlapping integration values were obtained from equations 5 and 7, respectively. In order to evaluate the Förster’s critical distance using equation 6, we have used *k*^2^ = 2/3; *Φ* = 0.15; *N* =1.36 for BSA [21, 22]. By using the equations 5–7, we have calculated the values of *E*, *r*, *R*_0_ and *J* and these values were found to be 0.28 nm, 1.9 nm, 1.63 nm and 7.15 × 10^−16^ cm^3^ m^−1^ for BSA-CBB system. In the present study, *r* is considered as the average value between the bound ligand and two tryptophan residues (Trp–135 and Trp–214) [23]. The value of *r* less than 7 nm indicated the non–radiative energy transfer between BSA and CBB [15].

### Effect of CBB on the Conformation of protein Synchronous fluorescence measurements

**Fig. 6.**
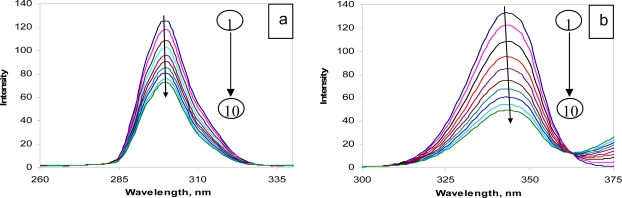
Synchronous fluorescence spectra of BSA: (a) Δλ = 15 nm; (b) Δλ = 60 nm. C_BSA_ = 2.5 μM (1), C_CBB_ = 2.5, 5.0, 7.5, 10.0, 12.5, 15.0, 17.5, 20.0 and 22.5 μM (2–10).

Synchronous fluorescence measurements were carried out in order to get the information on the molecular environment in the vicinity of the fluorophores (Tyr and Trp) of protein. Synchronous fluorescence spectra of BSA (Fig. 6) were obtained by simultaneously scanning the excitation and emission monochromater maintaing Δ*λ* = 15 nm (Tyr excitation) and Δ*λ* = 60 nm (Trp excitation) between them. Fig. 6 shows the effect of CBB on the synchronous spectrum of protein when Δ*λ* = 15 nm (Fig. 6a) or Δ*λ* = 60 nm (Fig. 6b). As it is evident, the intensity of the tryptophan and tyrosine decreased in the presence of CBB; but no significant shift was noticed in the signals. This supported the preferential quenching of Trp residues over Tyr residues. This indicated that the binding between CBB and the protein did not lead to a change in the polarity of the microenvironment of the tryptophan and tyrosine residues, however, the internal packing of the protein changed.

### UV–visible absorption measurements

We have recorded the absorption spectra of BSA in the absence and presence of CBB (Figure not shown). The absorption peaks observed around 280 nm were noticed to be shifted to the lower wavelength with increase in the concentration of CBB indicating the extension of the peptide strands of protein molecules [24, 25].

### FTIR measurements

**Fig. 8.**
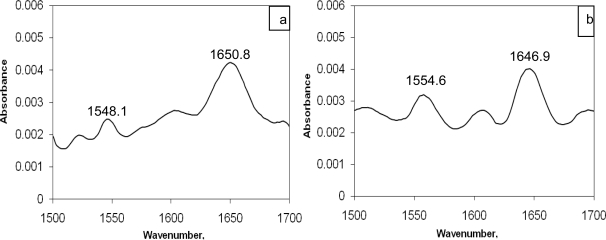
FTIR spectra and difference spectra of BSA: (a) free BSA (subtracting the absorption of the buffer solution from the spectrum of the protein solution) and (b) the difference spectra of BSA (subtracting the absorption of the CBB–free form from that of CBB–BSA bound form); C_BSA_ = C_CBB_ = 2.5 μM.

FTIR spectroscopy is a well defined tool for the determination of protein’s secondary structure [26, 27]. The conformational sensitivity of amide bands is governed by the two most important factors *viz.,* hydrogen bonding and the coupling between the transition dipoles. Both the amide I (noticed around 1653 cm^−1^ due to C=O stretching) and amide II bands (observed around 1548 cm^−1^ due to C–N stretch coupled with N–H bending mode) of the protein have the relationship with secondary structure of protein [28]. It is evident from Fig. 8a and Fig. 8b that the secondary structure of BSA was changed. The amide I band was noticed to be shifted from 1650.8 cm^−1^ (in free BSA) to 1646.9 cm^−1^ (in CBB–BSA) while amide II band was shifted from 1548.1 cm^−1^ (in free BSA) to 1554.6 cm^−1^(in CBB–BSA complex).

### Location of binding site

The principal regions of the ligand binding sites of albumin are located in the hydrophobic cavities of subdomain IIA and IIIA. Sudlow *et al* [29, 30] have suggested two distinct binding sites (site I and site II) in SAs. Warfarin, phenyl butazone *etc*., have high affinity towards site I while ibuprofen, flufenamic acid *etc*., exhibit affinity towards site II. Zhang *et al* [31] have suggested another site (site III) that has the affinity for digitoxin. In order to locate the binding site in BSA, we have recorded the fluorescence intensity of CBB–BSA in the presence and absence of site probe and in turn calculated the values of binding constant. The corresponding values are given in Table 2. It is clear from Table 2 that there is a significant decrease in the binding constant of CBB–BSA in presence of warfarin. However, the binding constant values remained almost same in presence of ibuprofen and digitoxin. These results revealed that the site I was the main binding site for CBB on protein. Therefore, the site I located in the hydrophobic pocket of subdomain IIA was proposed to be the binding site for CBB in protein.

## Conclusions

In this paper, the interaction between coomassie brilliant blue G and bovine serum albumin was investigated for the first time by fluorescence spectroscopy, UV–visible absorption and FTIR spectroscopy under simulative physiological conditions. The fluorescence data indicated the strong interaction as characterized by binding constant value of 4.20 × 10^4^ M^−1^ at 302 K. Further, the fluorescence quenching mechanism was observed to be dynamic process. Hydrophobic force played the major force in the interaction process.

## Figures and Tables

**Tab. 1.** K_SV_, K_q,_ binding and thermodynamic parameters of CBB–BSA.

**Temp. (K)**	***K***_***SV***_ **(mol^−1^)**	***K*** **(mol^−1^)**	***n***	***ΔG*^0^** **(kJ mol^−1^)**	***ΔH*^0^** **(kJ mol^−1^)**	***ΔS*^0^** **(J mol^−1^K^−1^)**
293	5.29 × 10^4^	2.81 × 10^4^	0.94	−24.96		
302	6.52 × 10^4^	4.20 × 10^4^	0.96	−26.73	48.0	2.49 × 10^2^
309	8.74 × 10^4^	7.95 × 10^4^	0.99	−28.99		

**Tab. 2.** The comparison of binding constants of CBB-BSA before and after the addition of site probes.

	**K without the site probe (mol^−1^)**	**K with warfarin (mol^−1^)**	**K with ibuprofen (mol^−1^)**	**K with digitoxin (mol^−1^)**
BSA	4.20 × 10^4^	7.73 × 10^3^	4.22 × 10^4^	4.19 × 10^4^
